# Pharmacokinetic and pharmacodynamic characterization of a novel formulation containing co-formulated interferons alpha-2b and gamma in healthy male volunteers

**DOI:** 10.1186/s40360-016-0103-8

**Published:** 2016-12-07

**Authors:** Idrian García-García, Ignacio Hernández-González, Alina Díaz-Machado, Carlos A. González-Delgado, Sonia Pérez-Rodríguez, Yanelda García-Vega, Rosario Campos-Mojena, Ángela D. Tuero-Iglesias, Carmen M. Valenzuela-Silva, Alieski Cruz-Ramírez, Alis Martín-Trujillo, Héctor Santana-Milián, Pedro A. López-Saura, Iraldo Bello-Rivero, for the CIGB-128-A Study Group, Ivonne Rivero-Vázquez, Laura Barrero-Viera, Maylén Álvarez-Delgado, Maura Tamayo-Rodríguez, Marlene David-Baldo, Lourdes Olivera-Ruano, Gricel González-Gamiz, Majel Cervantes-Llano, Reinier Hernández-Rodríguez, Leovaldo Álvarez-Falcón, Ivón Howland-Álvarez, Yolanda Cruz-Gómez

**Affiliations:** 1 Clinical Research Direction, Center for Genetic Engineering and Biotechnology, Ave. 134 b/23 and 25, Cubanacán, Playa, P.O. Box 6332, Havana, Cuba; 2 Isotope Center, San José de las Lajas, Mayabeque, Cuba; 3 National Center for Toxicology, “Carlos J. Finlay” University Hospital, Havana, Cuba; 4 Development Direction, Center for Genetic Engineering and Biotechnology, Havana, Cuba

**Keywords:** Interferons, Pharmacokinetics, Pharmacodynamics, Neopterin, Beta2-microglobulin, 2′–5′ oligoadenylate synthetase

## Abstract

**Background:**

More potent antitumor activity is desired in Interferon (IFN)-treated cancer patients. This could be achieved by combining IFN alpha and IFN gamma. The aim of this work was to characterize the pharmacokinetics and pharmacodynamics of a novel formulation containing a co-formulated combination of IFNs alpha-2b and gamma (CIGB-128-A).

**Methods:**

A group of nine healthy male subjects received intramuscularly 24.5 × 10^6^ IU of CIGB-128-A. IFN concentrations were evaluated for 48 h. Serum neopterin, beta2-microglobulin (β_2_M) and 2′–5′ oligoadenylate synthetase (2′–5′ OAS), classical IFN-inducible serum markers, were measured during 192 h by enzyme immunoassay and body temperature was used as pharmacodynamic variable as well.

**Results:**

Concerning pharmacokinetics, serum IFNs’ profiles were better fitted to a mono-compartmental model with consecutive zero order and first order absorption, one bioavailability value. No interferences by simultaneous administered IFNs were observed in their typical similar systemic profiles. Neopterin and β_2_M time profiles showed a delay that was efficiently linked to pharmacokinetics by means of a zero order absorption rate constant. Neopterin level was nine-fold higher than initial values, 48 h post-administration, an increment not described before. At this time, mean serum β_2_M peaked around the double from baseline. Serum concentrations of the enzyme 2′–5′ OAS was still elevated on the 8 day post-injection. The formulation was well tolerated. Most frequent adverse reactions were fever, headache, arthralgia and lymphopenia, mostly mild.

**Conclusions:**

The administration of co-formulated IFN alpha-2b and IFN gamma likely provides improved pharmacodynamic properties that may be beneficial to treat several malignancies.

**Trial registration:**

Cuban Public Registry of Clinical Trials RPCEC00000118, May 24, 2011.

## Background

Approaches to improve efficacy, tolerance and patient’s compliance and quality of life are permanent issues in pharmacological therapy. Some efforts have succeeded in favoring the bioavailability of the modified drug. Other actions are based on extending the systemic half-life through slower absorption.

Interferons (IFNs), like other low molecular weight protein drugs, have a relatively short half-life. Their conjugation to polyethylene-glycol (PEG) represented a favorable step forward to face this drawback [1]. Nevertheless, improved pharmacodynamics by the combination of two agents that have the potential to act synergistically can be another reasonable alternative. In that sense, IFN alpha-2b and IFN gamma have recognized synergistic antiproliferative effects on several tumor cell lines [2] based on the expression and activation of several IFN regulated genes [3]. The physical interaction between both IFN receptor complexes seems to be the first step for triggering intracellular signals that promote the potentiation of biological activities between both IFNs [4, 5].

The experience of the Center for Genetic Engineering and Biotechnology (CIGB, in Spanish) in the development of several formulations that contain recombinant molecules has allowed to obtain a novel formulation (CIGB-128-A) based on the combination of IFNs alpha-2b and gamma mixed in antiproliferative synergistic proportions defined in vitro [6]. The peri- and intralesional administration of the co-formulated IFNs was safe and effective for the treatment of elder patients with advanced, recurrent or resistant to previous treatments basal and squamous cell skin carcinomas [7]. In patients with mycosis fungoides, a similar IFN combination did not modify the kinetics of individual IFNs’ concentrations, but it produced a significant increment in neopterin serum levels, a well-known pharmacodynamic measure in IFN studies [8].

As part of CIGB-128-A biological characterization, it was necessary to carry out a clinical study in healthy male subjects to integrally analyze pharmacokinetics and pharmacodynamics, using available modeling tools. This population has a less inter-individual variability than oncologic patients. In order to describe the kinetic behavior of IFN-induced response, neopterin, β_2_-microglobulin (β_2_M), 2′–5′ oligoadenylate synthetase (2′–5′ OAS) as well as body temperature were used as pharmacodynamic variables.

## Methods

A phase one clinical trial was carried out at the National Center for Toxicology, in Havana, a certified reference unit for this type of studies.

### Subjects

Inclusion criteria were: no history of chronic diseases, no acute illness in the previous 30 days, no symptoms or signs at physical examination and laboratory tests, and no presence of HIV and hepatitis B and C virus infection markers in serum. Toxic habits, history of hypersensibility to any drug, treatment with any IFN formulation in the previous 6 months or with any drug in the previous 15 days, surgical intervention in the previous 6 months and blood donations in the previous 2 months were exclusion criteria. Subjects could withdraw from the trial voluntarily, due to occurrence of severe adverse reactions, or by the appearance of any exclusion criteria.

### IFN formulation

CIGB-128-A, Heber Biotec, Havana, a stabilized lyophilized powder formulation was used. Each vial contains 3 and 0.5 × 10^6^ IU of human recombinant IFNs alpha-2b and gamma, respectively, both produced in *E. coli*, trehalose, succinic acid and human serum albumin.

### Study design

Each volunteer received 24.5 × 10^6^ IU of CIGB-128-A intramuscularly, in the gluteus region. This represents 21 × 10^6^ IU of IFN alpha-2b and 3.5 × 10^6^ IU of IFN gamma. For this, the content of seven vials was diluted in 2 mL of water for injection. Detectable serum levels of both IFNs and their surrogate markers were expected at this dose level. The product was administered early in the morning after overnight fasting. Simultaneously with CIGB-128-A injection and thereafter, volunteers received oral antipyretic medication in order to mitigate flu-like symptoms produced by IFN. Volunteers were regularly checked for vital signs and adverse manifestations during the study. They were hospitalized during the first 48 h, thereafter ambulatory monitoring and blood sampling continued until 8 days.

### Laboratory evaluations

For IFN alpha-2b and IFN gamma measurements, samples were collected by venipuncture before injection and after 2, 3, 4, 6, 7, 8, 10, 12, 14, 16, 24, 36, and 48 h. Neopterin, β_2_M and 2′–5′ OAS determinations in serum were extended until 192 h after injection. These variables were assessed before injection and after 6, 12, 24, 48, 72, 96, 120, 168, and 192 h after. Some routine hematological (hemoglobin, platelet, leukocyte counts) and biochemical (hepatic enzymes, creatinine) determinations were evaluated for safety during the sampling period.

All the pharmacological variables were measured using commercially available enzyme immunoassay (EIA) kits, IFN alpha (Bender MedSystem, Germany, LOQ: 3.2 pg/mL, CV: 7.2 %), IFN gamma (Bender MedSystem, Germany, LOQ: 0.99 pg/mL, CV: 5.7 %), neopterin (HENNING test, BRAHMS Diagnostica, Berlin, Germany, LOQ: 0.5 ng/mL, CV: 6.9 %), β_2_M (Quantikine® IVD®, R&D System, UK, LOQ: 0.2 μg/mL, CV: 7.1 %) and 2′–5′ OAS isoform 1 (USCNK Life Science, USA, LOQ <1.5 ng/mL, CV < 12 %). In all cases, sera were stored at −70 °C until testing. Clinical laboratory variables were tested using advanced automated analyzers.

### Data analysis

Serum concentration versus time profiles of IFN alpha-2b and IFN gamma were analyzed using MONOLIX (Lixoft S.A.S, version 4.3.2, 2014, Paris, France). A previous evaluation showed the combined observational modeling as the most appropriated. A log-normal parameter distribution was selected, except for absorbed fraction (F), which was considered as logit-normal. Seven alternative compartmental models were evaluated, according with the number of compartments and the complexity of the absorption process. Table 1 describes models considered.

**Table 1:** Model description of the seven compartmental models used and estimated parameters

Model	Description	Parameters
I	Mono-compartmental with zero order absorption and bioavailability	Tk0, F, V, k
II	Mono-compartmental with first order absorption and bioavailability	ka, F, V, k
III	Bi-compartmental with first order absorption and bioavailability	ka, F, V, k, k_12_, k_21_
IV	Mono-compartmental with simultaneous zero order and first order absorption	Tk0, ka, F, V, k
V	Mono-compartmental with consecutive zero order and first order absorption. One bioavailability value	Tk0, ka, F, V, k
VI	Mono-compartmental with two simultaneous first order absorptions	ka1, F, ka2, V, k
VII	Mono-compartmental with consecutive zero order and first order absorption. One bioavailability value for each absorption component	Tk0, ka, F1, F2, V, k

*Tk0* duration of zero order absorption, *F* bioavailability associated to absorption processes, *V* volume of distribution of central compartment, *ka* rate constant of first order absorption, *k* first order elimination rate constant, k_12_ and k_21_: first order inter-compartmental rate constants

The objective function for model selection was the Akaike Information Criterion (AIC). Additionally, we considered the coefficient of correlation between individual predicted values and actual measured concentration.

Area under the curve (AUC) was calculated by means of the trapezoidal rule from measured individual concentrations and from model predicted concentration values. AUC relative prediction error also contains information about model adequacy and is calculated as follow.

$$ \mathrm{P}\mathrm{E}\left(\%\right)=\frac{{\mathrm{AUC}}_{\mathrm{p}}\hbox{-} {\mathrm{AUC}}_{\mathrm{m}}}{{\mathrm{AUC}}_{\mathrm{m}}}\times 100 $$

Where AUC_p_ is the area calculated from individual predicted concentrations and AUC_m_ is the area calculated from measured concentration values. As a measure of bias, we considered the mean relative prediction error and its root mean square as precision assessment [9].

We used the following relation for clearance calculation.

$$ \mathrm{C}\mathrm{I}=\frac{\mathrm{Dose}\cdot \mathrm{F}}{{\mathrm{AUC}}_{\mathrm{p}}} $$

An integrated pharmacokinetics/pharmacodynamics (PK/PD) model was written using the build-in MLXTRAN code in MONOLIX, based on the best-fit PK model and a classical indirect response model with response stimulation [10].

$$ \frac{\mathrm{dR}}{\mathrm{dt}}={\mathrm{k}}_{\mathrm{in}}^0\cdot \left(1+\frac{{\mathrm{S}}_{\max}\cdot {\mathrm{C}}_{\mathrm{p}}}{{\mathrm{S}\mathrm{C}}_{50}+{\mathrm{C}}_{\mathrm{p}}}\right)\hbox{-} {\mathrm{k}}_{\mathrm{out}}\cdot \mathrm{R} $$

Where R is the response, k^0^_in_ is the zero order rate constant for the production of response, k_out_ is the first order rate constant for response decrement and C_p_ is the serum concentration, S_max_ is the maximum stimulatory factor attributed to drug and SC_50_ is drug concentration producing 50 % of maximum stimulation.

**Fig. 1:**
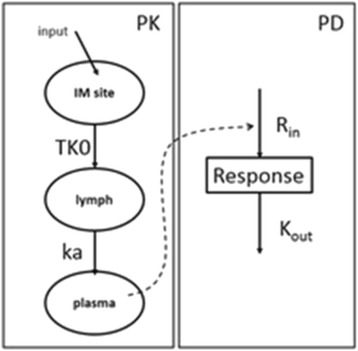
Schematic PK-PD structural model for IFN response

To account for differences in response between both IFNs related to pharmacokinetics, a delay equivalent to the duration of zero order absorption (Tk0) was introduced, manifest in the case of neopterin and β_2_M. For 2′–5′ OAS and body temperature, initial response level was added to the model. A diagram to illustrate the final adopted structural model is shown in Fig. 1.

Running integrated PK/PD model in MONOLIX, we left constant pharmacokinetic parameters and its standard deviation (fixed and random effects) as previously estimated, allowing the system to estimate individual pharmacokinetic parameters and population and individual pharmacodynamic parameters.

Description of pharmacokinetic and pharmacodynamic parameters were done using Microsoft Excel 2010 (Microsoft Corp.; USA) data analysis package. Categorical analysis (Wald’s test) was performed with MONOLIX. Vital signs, hematological counts and blood chemistry were analyzed using paired analysis (Student’s *t* test or Wilcoxon’s test) depending on the normality assumption, taking into account Bonferroni adjustment for multiple comparisons. SPSS for Windows version 15.0 was the software used for these safety analyses.

## Results

Nine healthy male volunteers received the tested formulation from a total of 19 subjects that were checked. This sample size was estimated using the method for 95 % confidence interval of a mean taken into account previous pharmacodynamic results obtained in patients with mycosis fungoides [8]. Skin white color prevailed (66.7 %). Their age ranged from 23 to 39 years-old (28 ± 5 years), weighed 52 to 98 Kg (71 ± 16 Kg) and were 162 to 175 cm (170 ± 5 cm) tall.

### Pharmacokinetic analysis

The best-fit model was number V for both IFNs. Table 2 shows AIC and correlation coefficient, while Table 3 summarizes the pharmacokinetic parameters estimated according to model V.

**Table 2:** Values of objective function and correlation between measured and predicted concentrations

Model	IFN	AIC	R^2^
I	alpha	1059	0.963
	gamma	1335	0.963
II	alpha	1160	0.897
	gamma	1375	0.940
III	alpha	1167	0.898
	gamma	1380	0.941
IV	alpha	1062	0.963
	gamma	1373	0.956
V	alpha	980	0.983
	gamma	1262	0.967
VI	alpha	1164	0.897
	gamma	1384	0.938
VII	alpha	1013	0.984
	gamma	1341	0.965

*AIC* Akaike Information Criterion

**Table 3:** Mean individual pharmacokinetic parameters and confidence intervals for IFN alpha-2b and IFN gamma (Model V)

Parameter (units)	Definition	IFN alpha-2b		IFN gamma	
		Mean	95 % CI	Mean	95 % CI
Tk0 (h)	Duration of zero order absorption	5.71	5.55–5.87	2.66	2.08–3.23
ka (h^–1^)	First order absorption rate	0.15	0.12–0.19	0.42	0.25–0.58
F	Bioavailability	0.22	0.15–0.30	0.15	0.15–0.15
V (L)	Volume of distribution of central compartment	38.4	38.4–38.5	118	21–214
k (h^−1^)	First order elimination rate order	0.21	0.18–0.23	0.09	0.08–0.09
Cl (L/h)	Total clearance	1.8	1.1–2.4	4.3	0.4–8.1

**Fig. 2:**
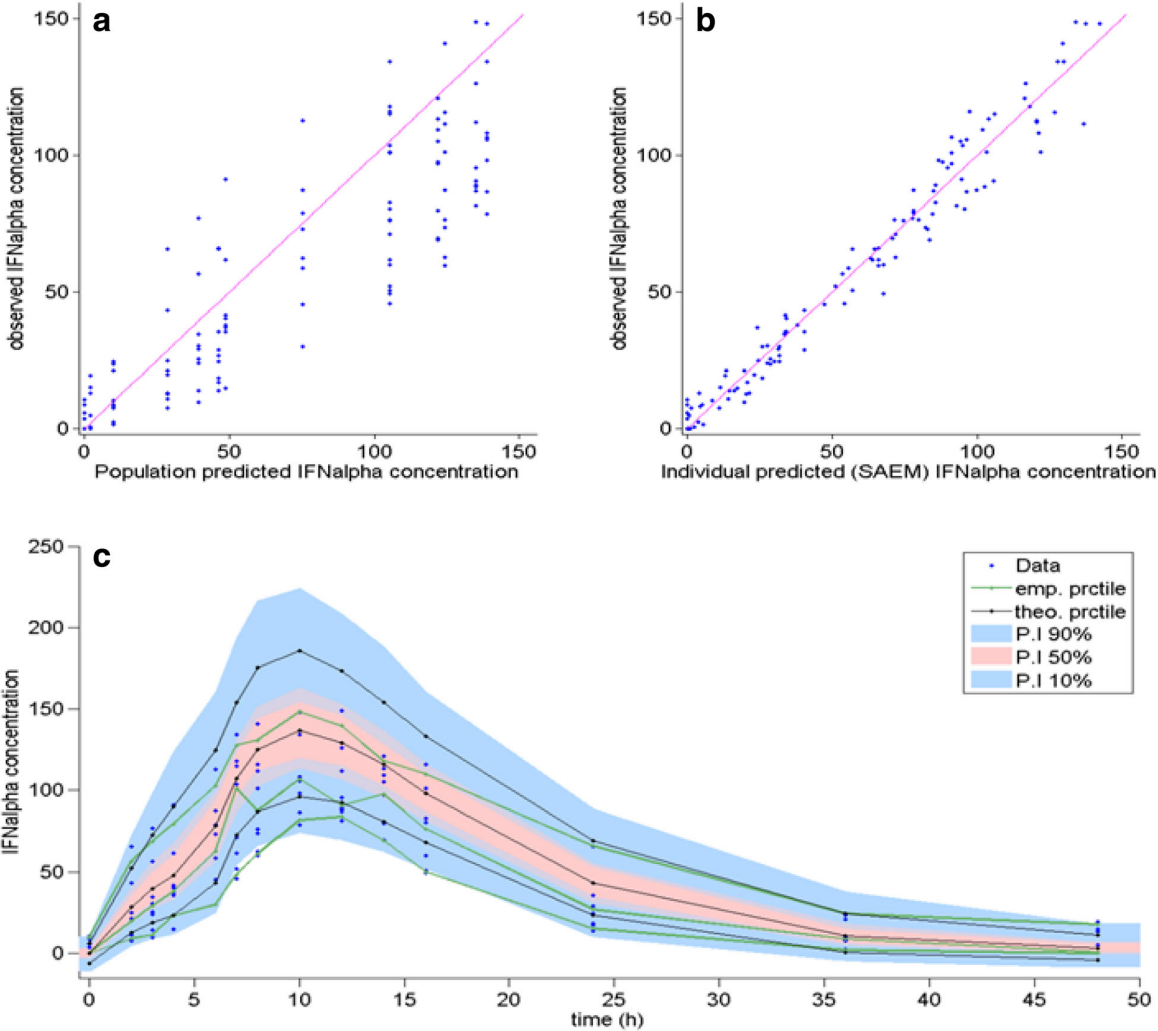
Relation between measured and predicted IFN alpha-2b concentrations in serum. Legend: Data correspond to IFN alpha-2b concentrations (pg/mL) measured by EIA after a single intramuscular administration of 24.5 × 10^6^ IU of CIGB-128-A to the nine healthy male subjects, according to model V. **a** Population predicted concentration. **b** Individual predicted concentration and (**c**) VPC graph

**Fig. 3:**
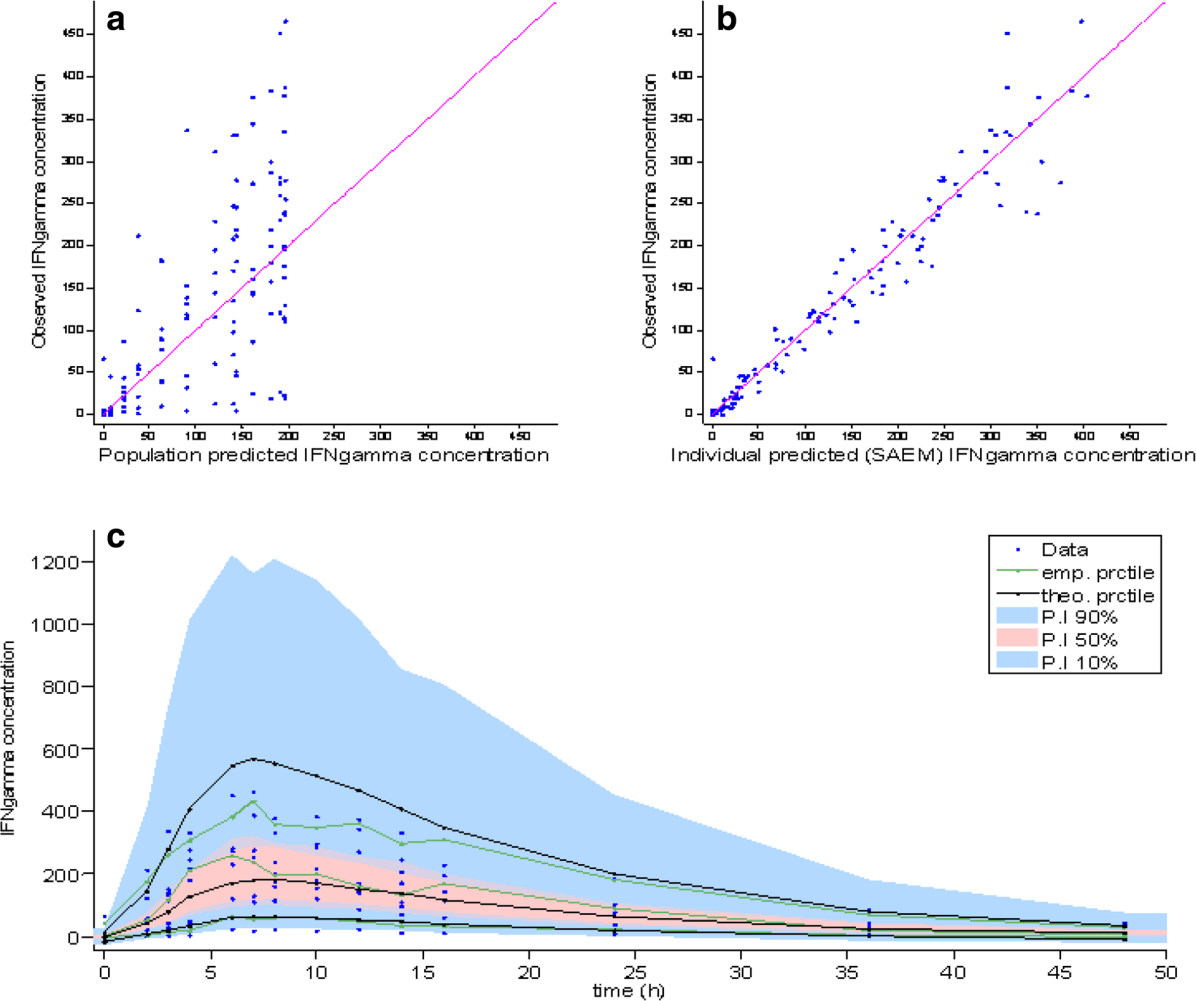
Relation between measured and predicted IFN gamma concentrations in serum. Legend: Data correspond to IFN gamma concentrations (pg/mL) measured by EIA after a single intramuscular administration of 24.5 × 10^6^ IU of CIGB-128-A to the nine healthy male subjects, according to model V. **a** Population predicted concentration. **b** Individual predicted concentration and (**c**) VPC graph

Figures 2 and 3 depict the relation between measured and predicted concentration as well as the visual prediction checking (VPC) according to model V for IFNs alpha-2b and gamma, respectively. Raw data is plotted together with VPC graph.

Interestingly, IFN gamma also fit well to model V, but with a shorter duration of the zero order absorption step (*p* < 1×10^−10^, Wald’s test) and a higher volume of distribution (*p* = 0.008, Wald’s test).

Average serum IFN profiles were qualitatively similar, although the maximum was reached 3–4 h before for IFN gamma. IFN gamma concentrations were higher, in agreement with the administered mass, calculated from their respective specific activities. Pre-dose serum IFN concentration was undetectable or very low. Most volunteers bordered or surpassed 100 pg/mL of IFN alpha and 200 pg/mL of IFN gamma.

**Fig. 4:**
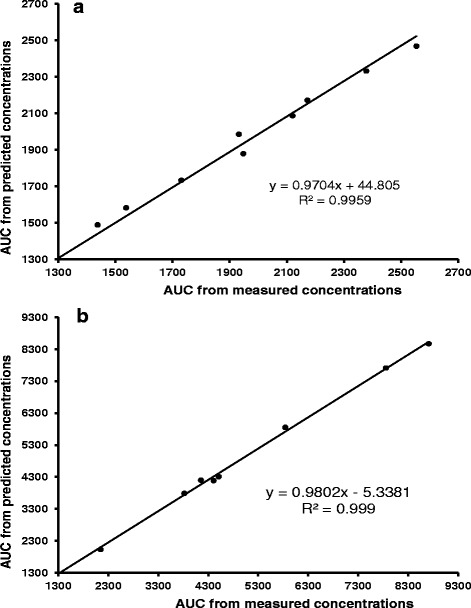
Correlation between actual and predicted AUC (pg · h/mL) for (**a**) IFN alpha-2b and (**b**) IFN gamma

Obtained AUC_48_ covered more than 96 % of AUC extrapolated to infinity. Table 4 shows areas under the curve of pharmacokinetic profiles for measured and predicted concentrations. Figure 4 represents the correlation between actual and predicted AUC for IFN alpha-2b and IFN gamma, respectively. It is noticeable that there is an excellent correspondence between actual and predicted AUC, which demonstrates the adequacy of best-fit model.

**Table 4:** Actual and predicted areas under the curve with its estimated bias and precision

IFN	AUC (pg · h/mL)		Bias 95 % CI	Precision 95 % CI
	Actual	Predicted		
alpha-2b	1979 (1751–2207)	1970 (1766–2174)	−0.1 (−1.8–1.6)	6.6 (3.4–9.8)
gamma	4655 (3068–6242)	4557 (3000–6114)	−2.0 (−3.5–-0.5)	9.5 (2.5 – 16.5)

AUC values are reported as mean (95 % CI)

### Pharmacodynamic analysis

Table 5 contains AIC and R^2^ for each measured response obtained from the application of the selected model. As observed, this model fits better in the case of IFN alpha than in the case of IFN gamma according to AIC.

**Table 5:** Criteria of integrated PK/PD model adequacy for each measured response

IFN	Criteria	Neopterin	β_2_M	2′–5′ OAS1	Temperature
alpha-2b	AIC	1218	1099	2122	1593
	R^2^	0.959	0.943	0.969	0.834
gamma	AIC	1569	1334	2283	1721
	R^2^	0.952	0.946	0.931	0.839

*AIC* Akaike Information Criterion

Table 6 summarizes pharmacodynamic parameters and its variability expressed as confidence intervals. Both IFNs were co-administered; therefore, the response due to each IFN cannot be separated. Nonetheless, according with our results IFN alpha-2b fits better to the selected PK/PD model. Only for body temperature, a similar increase and decrease in the response is observed when each IFN is separately modeled.

**Table 6:** Mean individual pharmacodynamic parameters and confidence intervals for IFNs alpha-2b and gamma

Parameter (units)	IFN alpha-2b		IFN gamma	
	Mean	95 % CI	Mean	95 % CI
Neopterin				
S_max_	25.8	25.2–26.4	16.7	15.8–17.6
SC_50_ (pg/mL)	15.5	14.8–16.1	19.5	13.8–25.3
k_in_ (h^−1^)	0.027	0.024–0.030	0.03	0.026–0.034
k_out_ (h^−1^)	0.022	0.022–0.022	0.0247	0.0247–0.0248
β2-microglobulin				
S_max_	1.03	0.95–1.1	4.5	3.81–5.1
SC_50_ (pg/mL)	0.31	0.03–0.58	76.4	37.2–115.7
k_in_ (h^−1^)	0.141	0.133–0.149	0.04	0.033–0.038
k_out_ (h^−1^)	0.070	0.067–0.073	0.0194	0.0190–0.0198
2′–5′ oligoadenylate synthetase				
S_0_ (ng/mL)	33.9	14.5–53.3	762	721–803
S_max_	25.8	10.6–41.0	104	39–169
SC_50_ (pg/mL)	419	392–446	730	394–1066
k_in_ (h^−1^)	1990	1754–2226	237	188–285
k_out_ (h^−1^)	28.20	14.21–42.18	17.7	6.2–29.1
Body temperature				
S_0_ (°C)	35.9	35.9–35.9	36.2	36.0–36.2
S_max_	9.7	9.5–9.8	7.2	6.7–7.6
SC_50_ (pg/mL)	252	239–264	374	254–493
k_in_ (h^−1^)	0.864	0.858–0.870	0.851	0.848–0.855
k_out_ (h^−1^)	1.0155	1.015–1.016	1.021	1.021–1.021

*S*_*0*_ baseline response, *S*_*max*_ maximum stimulatory factor attributed to drug, SC_50_: drug concentration producing 50 % of maximum stimulation, *k*_*in*_ the zero order rate constant for the production of response, *k*_*out*_ the first order rate constant for loss of response

**Fig. 5:**
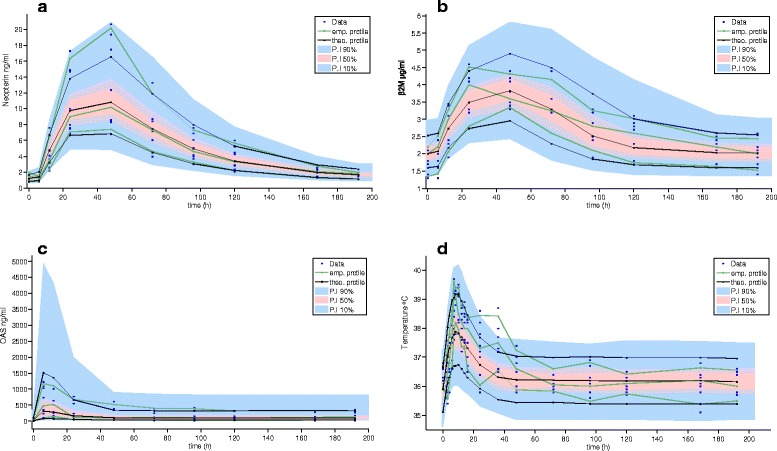
Model validation and raw data of pharmacodynamic variables for IFN alpha-2b. Legend: Data correspond to the nine healthy male subjects who received 24.5 × 10^6^ IU of CIGB-128-A at time 0. **a** Neopterin. **b** β_2_M. **c** 2′–5′ OAS1 and (**d**) Body temperature (first 48 h). Raw data is plotted together with VPC graph

**Fig 6:**
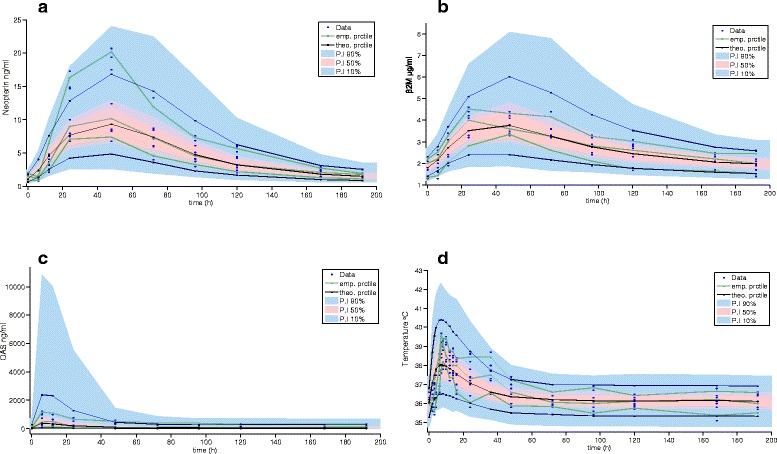
Model validation and raw data of pharmacodynamic variables for IFN gamma. Legend: Data correspond to the nine healthy male subjects who received 24.5 × 10^6^ IU of CIGB-128-A at time 0. **a** Neopterin. **b** β_2_M. **c** 2′–5′ OAS1 and (**d**) Body temperature (first 48 h). Raw data is plotted together with VPC graph

Figures 5 and 6 depict the VPC according to the selected integrated PK/PD model for IFNs alpha-2b and gamma, respectively. It is evident the adequacy of the integrate PK/PD model for IFN alpha-2b with neopterin and β_2_M, because Tk0 is almost equivalent to response initial delay, shorter in the case of IFN gamma.

Baseline levels of biomarkers were within normal values specified by the EIA kits suppliers. A strong neopterin production was obtained with co-formulated IFNs. An approximately nine-fold average increase was obtained 48 h after CIGB-128-A administration (Figs. 5a and 6a). Neopterin values remained clearly upper baseline levels between 12 and 120 h. All subjects increased this marker at least by six times compared to pre-dose values.

Average β_2_M approximately doubled the initial value between 24 and 48 h after injection, and then slowly returned to baseline at the end of the sampling period (Figs. 5b and 6b). Maximum levels of 2′–5′ OAS1 were achieved 6–12 h after CIGB-128-A administration; these were around 18-fold higher than baseline. Twelve hours later, OAS concentration was reduced, but levels remained stable from 96 h on, being approximately four-fold higher than baseline (Figs. 5c and 6c).

### Safety data

All volunteers presented at least one adverse event, predominantly flu-like symptoms, but none of them withdrew from the trial due to this or other causes. Fever and headache were registered in all subjects. Other manifestations such as arthralgias, lymphopenia, tachycardia, myalgias, malaise, tremors, anorexia and thrombocytopenia occurred in more than half of the individuals. Most events were mild (85.9 %), being well solved. No severe adverse events were recorded.

Despite the antipyretic treatment, the increment in body temperature was quite remarkable, reaching 39.0 °C in eight subjects. Peak temperature values were reached between 7 and 10 h after IFN administration (Figs. 5d and 6d).

White cells and platelet counts were reduced below normal values in most subjects. Particularly, mean changes from baseline in lymphocyte count were significant (*p* < 0.006) at several times, outstandingly 24 h post-injection, when the mean value was 0.7 × 10^9^/L. Other clinical laboratory measures were not significantly affected.

## Discussion

An initial zero order absorption rate, Tk0, was also observed by other authors after subcutaneous administration of IFN alpha [11]. As both IFNs are co-administered in a single formulation, we cannot differentiate their individual participation in the total pharmacodynamic responses. In addition, we cannot explain the high levels of a pharmacodynamic marker in blood because of the lack of empirical data to “fill” compartments defined in complex structural models based on mechanistic approaches. This situation is very common in clinical trials [12], like the present study.

Pharmacodynamics of CIGB-128-A formulation was characterized through three well-known IFN response markers, mediators of their main biological actions, traditionally used in this type of studies [13, 14]. Neopterin is an excellent marker of the immune-mediated cytotoxicity and induction of apoptosis in human malignancies [15]. Beta2-microglobulin plays a key role in tumor growth control and metastases [16]. The 2′–5′OAS enzyme interferes in the progression of the cell cycle [17] and produces degradation of viral RNA [18].

The delayed irruption of neopterin and β_2_M responses is commonly explained as a consequence of the complex intracellular pathways triggered after IFN binding to its surface receptor. This concept was successfully applied to the stimulation of the synthesis of the antiviral Mx protein by IFN alpha-2a subcutaneous administration to healthy volunteers [19].

Working with the obtained data, delay in neopterin and β_2_M responses could be related to Tk0. Existence of zero order absorption has been suggested to occur owing to saturated absorption just after administration [11]. Differences in molecular structure, consequently in physicochemical characteristics, can be a plausible cause for differences in absorption between IFNs alpha and gamma.

However, a delay was not observed for the other measured responses (2′–5′ OAS, temperature) which could obey to different triggering mechanisms [20]. Quantification of serum or plasma 2′–5′ OAS by EIA (instead of intracellular expression or enzymatic activity) to evaluate pharmacodynamics in humans is not easily identified in literature. Body temperature was used as pharmacodynamic variable as well, although its increment constitutes an undesired effect. IFN alpha induces fever, at least partly due to direct effects on the hypothalamic thermosensitive neurons, which involve also the opiate receptor mechanism [21]; while IFN gamma stimulates the release of pyrogenic interleukin-1 [22].

On the other hand, there are no data indicating interferences in the pharmacokinetic profiles of IFNs alpha-2b and gamma when they are simultaneously administered. Our findings reveal a pharmacokinetic behavior of IFN alpha-2b quite similar to that reported by other authors [11]. Distribution to a wide range of tissues and organs and a very fast clearance are distinctive characteristics of IFNs [23–27].

Despite IFNs antitumor activity being well-known at present, no major advances have been achieved in the treatment of solid and hematological malignancies in the last decades. An enhanced survival or a better objective response cannot be achieved by a dose increment since more severe adverse reactions could irrupt. Additionally, a more prolonged schedule reduces patient’s compliance.

A sustained full IFN-receptor interaction that triggers strong antiproliferative activities is desired in the cancer treatment with this cytokine. The slow systemic clearance of PEG-IFN alpha structures had led to less dosing, more efficacy and less toxicity in patients with chronic viral hepatitis [28]. However, pegylation produces a notable reduction in IFN bioactivity [29], probably due to a non-optimal interaction with its receptor. Additionally, high molecular weight pegylated IFNs could have more difficulties to penetrate into the tumor microenvironment and have an effective interaction with its receptor.

The combination of co-formulated IFNs alpha and gamma could improve antitumor effects of separate IFNs. CIGB-128-A formulation was developed on the basis of two essential criteria; first, the in vitro and in vivo potentiation of common biological activities of IFN alpha-2b and IFN gamma in certain proportions previously defined [6, 30] and second, their similar pharmacokinetics.

The high increments in serum neopterin levels in healthy subjects could support the hypothesis on which CIGB-128-A formulation was conceived. That nine-fold neopterin increase has not been reported with any subtype or variant of IFN. After a single intramuscular dose of 18 × 10^6^ IU IFN beta to healthy volunteers, serum neopterin increments were five-fold higher than baseline levels [13], leading to a lower S_max_ in a later modeling analysis [31]. Single subcutaneous doses of 27 and 36 × 10^6^ IU of IFN alpha-2a to healthy volunteers only produced four-fold increments in plasma neopterin concentrations [32]. The induction of neopterin by PEG-IFN alpha in patients or healthy volunteers only approximately tripled the initial values, 48 h after injection [14, 33, 34]. Pegylation seems to produce a reduction in S_max_ as occurred with Mx protein [19]. In the previous clinical trial in patients with mycosis fungoides, intramuscular 23 × 10^6^ IU of the co-formulated IFNs produced neopterin increments that were six-fold higher at the same sampling time [8].

Beta2-microglobulin increments are around 60 % after administration of PEG-IFN alpha [14, 34], lower than 100 % found 24–48 h after CIGB-128-A injection. Although this last increment was reached in a similar population after administration of 20 ×10^6^ IU of IFN alpha-2b [27], a slower return to initial levels was now observed.

Six hours after a single CIGB-128-A intramuscular injection, 2′–5 OAS1 serum levels were extensively increased in healthy volunteers. In IFN-treated patients, enzymatic activity of 2′–5 OAS appears to increase since 6 h and maintains elevated levels until 4–8 months later during chronic treatments [35]. OAS induced by CIGB-128-A continued detectable until 192 h and the moment to return to normality could not be predicted.

Nevertheless, we need to deepen into the mechanisms that trigger all these responses allowing us to explain the postulated enhanced effect. There are other factors making this task difficult, mainly those related to administered doses, like response saturation [32].

Pharmacodynamic findings were part of the overall information that justified approval of further clinical trials with CIGB-128-A formulation. In these trials more spaced dosing schedules are evaluated in patients with advanced or metastatic solid tumors without other therapeutic options. These are ongoing trials and preliminary results are encouraging.

The formulation was well tolerated. As expected, flu-like symptoms and hematological count reductions were the most common adverse events generated after administration. Bone marrow depression produced after only one IFN dose can be explained by increase in cortisol levels [36].

## Conclusions

In this work PK/PD of CIGB-128-A formulation, containing IFNs alpha 2b and gamma co-formulated in synergistic proportions, was characterized. The PK best-fit model was number V for both IFNs and average serum IFN profiles were qualitatively similar. Compared with literature data CIGB-128-A promotes strong neopterin and 2–5 OAS production and extends upper baseline levels until 98 and 120 h, respectively. A unique dose of more than 20 MIU of CIGB-128A was safe with no severe event reported. These properties indicate that this formulation is a good candidate to treat some oncologic diseases, without pharmacokinetic interferences or additional toxicity.

## Abbreviations

2′–5′ OAS: 2′–5′ oligoadenylate synthetase
AIC: Akaike Information Criterion
AUC: Area under the curve
CI: Confidence intervals
Cl: Clearance
EIA: Enzyme immunoassay
IFN: Interferon
PD: Pharmacodynamics
PEG: Polyethylene–glycol
PK: Pharmacokinetics
β_2_M: Beta2-microglobulin
